# Portrayal of the deep sea: Haeckel’s *Atolla wyvillei*, the challenger expedition, and the boundaries of science and illustration

**DOI:** 10.1007/s00114-026-02154-8

**Published:** 2026-09-14

**Authors:** Bernhard L. Bock

**Affiliations:** Institute for Zoology and Evolutionary Research, Friedrich Schiller University Jena, Erbertstraße 1, 07743 Jena, Germany

**Keywords:** Museum, Collection, Marine, Jellyfish, Medusa, Type

## Abstract

The *Challenger* expedition marked a turning point for marine biology, revolutionizing the scientific research of ocean life. It yielded a report of 50 volumes totaling nearly 30,000 pages—a monumental result that remains unchallenged. Among its vast contributions are reports by Ernst Haeckel on previously unknown species of radiolarians and medusae, which stand out for their lasting influence on the taxonomy of marine organisms. *Atolla wyvillei* Haeckel, 1880 is among these newly described medusae, named in honor of Sir Charles Wyville Thomson, the chief scientist of the HMS Challenger. Housed in the holdings of the Phyletisches Museum Jena (PMJ), the type specimen of *A*. *wyvillei* is carefully reexamined and documented with high-resolution imaging here for the first time. Furthermore, previously unknown microscope slide sections of an additional syntype of *A*. *wyvillei* are revealed. By directly comparing these primary physical preparations with Haeckel’s published plates, this study provides empirical evidence to resolve long-standing historical debates regarding Haeckel’s reliance on artistic interpretation or fleeting species descriptions. This physical reevaluation demonstrates that his illustrations were grounded in meticulous histological analysis, thereby providing an objective basis to reassess Haeckel’s taxonomic legacy and safeguard primary type material for contemporary and future research.

**Supplementary Information:**

The online version contains supplementary material available at https://doi.org/10.1007/s00114-026-02154-8.

## Introduction

For centuries, the global ocean’s depths remained an impenetrable abyss, largely dismissed as a lifeless void. Back in the early 19th century, natural science lacked both the technological capacity and the empirical mandate to systematically investigate the benthic layers of the deep sea. Scientific interest was rekindled in no small measure by contemporary attempts to connect the world via undersea communication cables, ranging from early telegraph lines to the modern internet. When retrieving failed cables from depths exceeding a thousand fathoms, engineers found them encrusted with diverse marine life (Broad 1998), proving that organisms could thrive in cold, deep waters. Concurrently, early marine dredging began, revealing that the seafloor was not merely a desert of mud, but a repository of extraordinary geological, zoological and mineral wealth—most notably symbolized by the discovery of polymetallic nodules, that were first discovered by the famous *Challenger* expedition (1872–1876) (Thomson 1873) (Belkin et al. 2021). The *Challenger* expedition was the first scientific expedition to explore the deep sea on a global scale (Packer & Paver, 2011; Zuroski 2016), and the first expedition to successfully map the physical, chemical, and biological characteristics of the global deep sea.

Alongside nearly 250 sailors, engineers, carpenters, and naval officers, a small scientific team (and a dog) comprised the crew. At more than 500 stations spread across nearly 80,000 miles, the crew measured depths, temperatures and collected water samples. The seafloor was dredged to sample marine life and geology, with specimens stored in vials and casks that were later distributed to various international scientists (Jones 2022). The results of the expedition were astonishing and remain deeply significant today. The crew’s efforts to map the seabed revealed that the oceans are not deepest in their center, as had previously been assumed. This knowledge eventually became indispensable to the concept of seafloor spreading, which is crucial to our modern understanding of plate tectonics (McNutt 2002). Furthermore, the scientific crew discovered both the deepest oceanic trench on Earth, the Mariana Trench, and the deepest known point of the seabed, the Challenger Deep. They also displayed extraordinary foresight by collecting micrometeorites from the deep sea, employing magnets for the first time to sample dredged sediments (Suttle et al. 2021), while compiling the world’s oldest collection of these tiny (< 1 mm) cosmic fragments (Ashworth 2022). The expedition has also become relevant in entirely unexpected ways, for example, the baseline historical samples collected by the crew are now critical to understanding how modern ocean acidification driven by climate change is impacting global marine ecosystems (Gebbie and Huybers 2019).

From a biological perspective, their most critical achievement was permanently disproving Edward Forbes’s (1815–1854) azoic hypothesis (Anderson and Rice 2006), which posited that no life could exist below 300 fathoms. Instead, they uncovered an immense diversity of deep-sea life. The more than 100,000 samples and specimens collected yielded a massive 50-volume report published between 1885 and 1895, containing nearly 30,000 pages by more than 75 contributing authors, describing around 4,700 new species. A set of these samples was sent to Jena to the famous German zoologist Ernst Haeckel (1834–1919), who was already recognized as a leading authority in marine zoology. Haeckel published extensive, heavily illustrated monographs on *Radiolarians* and *Deep Sea Medusae* (1881, 1887), describing dozens of novel species that were subsequently stored in the collection of the Zoological Institute at the University of Jena. However, his taxonomic methodology and artistic illustrations sparked intense debate both during his lifetime and in post-hoc scholarship. Critics manly argued that he fabricated non-existent taxa, lost primary type material, or produced inaccurate illustrations rendered useless for scientific inquiry (cf. Rütimeyer, 1868; His, 1874; Wells 1999; Straehler-Pohl et al., 2022). To address these historical critiques, this study examines a surviving original specimen and details Haeckel’s meticulous analytical methods, anchored by the identification of a newly designated syntype. Ultimately, this work overhauls the taxonomic status of these reevaluated species, making them fully accessible for contemporary research and contributing to the long-term preservation and curation of these primary type specimens.

### Historical background of the *Challenger* expedition

#### Sir Wyville Thompson

Upon entering St. Michael’s Parish Church in Linlithgow, an imposing stained-glass window by Clayton & Bell in the apse captivates the visitor’s gaze. It depicts four angels at the top, followed by scenes of humanity producing goods and representations of various animals. Beneath this scene, a ship sails across the sea, passing sharks, fish, birds, and whales above a floor of corals, shells, and crabs. The inscription reads: “IN MEMORY OF SIR CHARLES WYVILLE THOMSON OF BONSYDE PROF. NATURAL HISTORY UNIV. EDINBURGH DIRECTOR OF THE CHALLENGER EXPEDITION ERECTED BY HIS FRIENDS AND COLLEAGUES 1885”. Wyville Thomas Charles Thomson (1830–1882) was born on March 5, 1830, at Bonsyde, his family’s small estate. Early in life, he received elementary lessons in geology and botany from a Mr. Chalmers, followed by the formal study of botany and zoology upon entering university. He was appointed professor of botany in 1851 at the University of Aberdeen, professor of natural history in 1853 at Queen’s College (Cork, Ireland) and held the chair of natural history at the Royal College of Science in Dublin from 1868 to 1870. From 1870 onward, he held the prestigious chair of natural history at the University of Edinburgh.

Since the beginning of Sir Wyville’s studies, he made urgent requests to establish collections in public institutions and to investigate natural objects themselves, rather than merely trusting the reported observations of others. On May 30, 1868, Thomson addressed a letter to his friend William Carpenter (1813–1885) concerning living organisms dredged from 200 to 300 fathoms by Peter C. Asbjørnsen (1812–1885), and similar specimens dredged by Michael Sars (1805–1869) two years prior. As planned, Carpenter wrote to General Edward Sabine (1788–1883), President of the Royal Society, on June 18, 1868, explaining how he and Thomson could expand upon these highly interesting findings with minimal financial backing from the Admiralty. The proposal was immediately approved by the Council of the Royal Society that same day. By July 14, the Lords Commissioners of the Admiralty issued orders to outfit a vessel to meet the Royal Society’s requests (Thomson 1873; Redfern 1888). This initiative resulted in three oceanographic cruises aboard the *Lightning* (1869) and *Porcupine* (1869, 1870).

These successful investigations elevated Thomson’s scientific reputation, leading to his election as a Fellow of the Royal Society in 1869. He published the results of these three voyages in his book *The Depths of the Sea* on December 2, 1872, just 19 days before embarking on his most vital and fateful final voyage, the *Challenger* expedition. It was primarily through the initiation of Carpenter and Thomson, united by their mutual interest in deep-sea organisms, that the *Challenger* expedition was organized to follow up on their previous work. Given Thomson’s expertise and the specific aims of the expedition, investigating the physical conditions of the deep sea, determining the chemical composition of seawater, ascertaining the physical and chemical characteristics of benthic deposits, and examining organic life, he was selected as the chief scientist of the global voyage at the age of 42. Shortly after the *Challenger* returned on May 24, 1876, he was knighted in June, appointed Director of the Challenger Expedition Commission, and awarded the Royal Medal by the Royal Society for his outstanding leadership and scientific investigations.

While some preliminary data were reported during the voyage, the majority of the material had to be processed after their return under Sir Wyville’s general guidance. During these years, Thomson faced criticism for his decision to send some of the collected material to foreign specialists rather than keeping it entirely within Britain. Further, he and Carpenter were accused of plagiarism, a charge that was never proven (Rice 1976). In 1877, after laboring over an immense volume of specimens and data, Thomson published a two-volume general review titled *The Voyage of the “Challenger”: The Atlantic*. This publication generated exceptional public and professional interest, not only for its scientific conclusions but also for its beautifully executed illustrations. Throughout the text, he explicitly recognized the valuable assistance of his colleagues and the expedition staff (Redfern 1888).

However, the psychological stress and physical strain of continuously working to publish the final reports severely damaged his health, and he never fully recovered from a serious illness in 1879 Haeckel E (1879). His declining health forced him to resign as director of the Challenger Expedition Commission in 1881, where he was succeeded by John Murray. Unlike Murray, Sir Wyville never witnessed the completion of the final reports, as he died on March 10, 1882, at Bonsyde, leaving behind his wife and son. Alongside his many elected fellowships, including the F.R.S.E. (1855), M.R.I.A. (1861), and F.R.S. (1869), as well as memberships in the Linnean, Geological, and Zoological societies, he received several honorary degrees, including a Doctor of Philosophy *honoris causa* from the University of Jena in 1877. This occurred during the year Ernst Haeckel served as dean of the faculty. Haeckel undoubtedly influenced the decision to grant him this honorary degree and personally penned the entry into the *Dekanatsbuch* University Archives Jena (n.d.).

#### Haeckel and his way to the challenger

Only a few points concerning Germany’s most famous and controversial zoologist, Ernst Heinrich Philipp August Haeckel, will be highlighted here. In 1856, Haeckel made his first visit to Villefranche-sur-Mer, where he conducted his earliest observations of living radiolarians—a group of single-celled organisms enclosed in a silicate, glass-like exoskeleton (Dolan 2019). He later visited Messina (1859/1860) and Naples (1859) to study this “inexhaustible well of the finest enjoyment” (Haeckel 1862), which eventually culminated in one of his most remarkable scientific and, so said, artistic accomplishments: his first monograph, *Die Radiolarien: eine Monographie* (1862). This and subsequent works, including the spectacularly illustrated *Rüsselquallen* (1864), the seminal *Generelle Morphologie der Organismen* (1866), the widely discussed *Natürliche Schöpfungsgeschichte* (1868), as well as *Die Siphonophoren* (1869) and *Die Kalkschwämme* (1872), established his fame at a young age and made him a prominent figure in marine science (Dolven et al. 2014; Hoßfeld 2012; Levit & Hoßfeld, 2019; Dolan 2019). Leveraging his newly gained popularity and reputation, Haeckel received two decisive letters that guided his path toward the *Challenger* project. The first came from Edwin Ray Lankester (1847–1929), who suggested: “[t]his year it would be a good one for you to come to the British Association. It meets at Glasgow. The naturalists of the Challenger will be there and many of their treasures. September 5th is the date. Will you come? You will be treated with special honour and distinction by the University & citizens of Glasgow, and can not fail to enjoy the occasion.” (Lankester 1876; EHH Jena, A 27311). The second letter was sent by Paul Rottenburg (1846–1929) on August 3, 1876, informing Haeckel of the British Association meeting: “with sincere regret I miss[ed] the name “Haeckel” in the list of strangers expected at this year’s meeting, and I hereby take the liberty of asking you in particular to join. It is a more than justified national pride that makes me wish to see you at the meeting, and sincere reverence if I ask you to be my guest in the event of your stay. – As a man and champion of science, you almost owe a visit to Glasgow and Scotland in my eyes; – a country so badly biased by dogma and doctrine, it is more than necessary that the gospel of “der natürlichen Schöpfungs Geschichte” should be preached here. *–* “(Rottenburg 1876a, b; EHA Jena, A 29934). Earlier invitations to the meeting received by Huxley in 1865 and 1866 had been declined by Haeckel (EHA Jena, A 19319; EHA Jena, A 19318; British Association for the Advancement of Science (1867). This time, however, circumstances were different and he appears to have answered immediately, as Rottenburg replied on August 15, expressing the delight he and his wife felt regarding Haeckel’s agreement on the visit (Rottenburg 1876a, b; EHA Jena, A 19995).

In the autumn of 1876, Haeckel received a letter from Sir Wyville Thomson asking him to write a few explanatory lines for officials regarding the importance and condition of the *Challenger* material, noting that they highly valued the “opinion of a well-known foreigner!” and adding that he would send him “his Radiolarians as soon as possible” (Thomson, 1876; EHA Jena, A 18090). Haeckel accepted the invitation immediately, recognizing the immense potential of the collection and the fact that many of the era’s most renowned scientists would be collaborating on it. While attending the British Association meeting in September 1876, that he privately found “least satisfying” (suggesting Lankester may have exaggerated the appeal), he met with Wyville a few days later, gaining firsthand access to the fabulous specimens collected by the crew (Haeckel 1876a, b; EHH Jena, A 38464). Rottenburg’s invitation and this subsequent meeting provided the initial spark for Haeckel to author four monumental *Challenger* reports: *Deep-Sea Medusae* (1881), the before mentioned *Radiolaria* (1887), *Siphonophora* (1888), and *Deep-Sea Keratosa* (1889). While this could be viewed as a fortunate coincidence, the correspondence shows that these highly influential figures were highly determined to get Haeckel on board for the project, which he ultimately accepted.

Haeckel did not merely coin foundational terms such as *ecology*, *phylogeny*, or *stem cell* (*Stammzelle*) (Haeckel 1866, 1868; cf. Bölsche 1906 and Ramalho-Santos et al., 2007) for which he receives widespread attention today. Unfortunately, the same degree of attention is rarely paid to his *Challenger* Reports, which represent some of the most underrated morphological treatises on marine invertebrates. Inseparably interwoven with his *Deep-Sea Medusae* report is his *Monographie der Medusen* (1879, 1880). In the latter text, Haeckel occasionally provided only brief species diagnoses (e.g., 1880, pp. 442, 487, 488–489), stating that “a detailed description and illustration follows in the ‘Deep-sea Medusae of the *Challenger* Expedition”, published in 1881. Haeckel’s pioneering contributions laid the foundation for decades of evolutionary research and remain highly relevant today, as exemplified by his comprehensive taxonomy of radiolarians (O’Dogherty et al. 2021), his foundational anatomical concepts (Richardson et al., 2002), as well as the first modern systematic monograph on Scyphomedusae (Russell 1953). In total, Haeckel contributed an astonishing 2,790 pages and 230 plates, comprising nearly 10% of the entire 50-volume publication of the *Challenger* report (Murray 1895). His plates are among the most recognizable of the entire expedition (Dolan 2024). Although copies (about 750) of the *Challenger* reports were distributed globally to key institutional libraries, detailed engagement with their specialized scientific content was historically restricted to a relatively small circle of selected specialists (Hedgpeth 1946; Burstyn 1975). Despite this, the general public has encountered these images in one form or another, as Haeckel drew heavily upon these illustrations to create his famous *Kunstformen der Natur* (1904). Many of these plates were inspired by or directly adapted from the *Challenger* volumes (Evans et al. 2016). The cultural influence of *Kunstformen der Natur* extended far beyond biology, leaving a major mark on architecture, art, and inspiring prominent figures like Kandinsky, Klimt, and Munch (e.g., Stauffer 1957; Cordulack 2002; Proctor 2006; Crowther et al., 2012; Halpern & Rogers, 2013; Ricci 2019; Levit & Hoßfeld, 2019). Yet, while praised as an artistic masterpiece, it is often overlooked that *Kunstformen der Natur* was designed as a scientific publication. This misunderstanding is reflected in the fact that most modern reproductions completely omit the original text volume containing roughly 250 pages of species designations, remaining difficult to find (Dolan 2024).

As recently reemphasized by John Dolan (2024), the skill of Haeckel’s friend and lithographer, Adolf Giltsch (1853–1911), was instrumental in granting Haeckel’s work such prominence and extraordinary longevity in the collective consciousness. In the preface to his *Report on the Radiolaria* Haeckel E (1887), Haeckel explicitly states: “the figures of new species of Radiolaria (about 1600 in number) which appear in the atlas of one hundred and forty plates accompanying this Report, were nearly all drawn with the camera lucida, partly by Mr. Adolph Giltsch and partly by myself”. Haeckel held Giltsch’s work in high esteem, playfully appointing him *Doctor Artis*,* honoris causa* and noting that their 12 years of collaborating on the *Challenger* material comprised some of the most precious memories of his life (Haeckel 1911; EHA Jena, A 51444). As previously noted, Haeckel attracted, and continues to attract, both significant praise and sharp condemnation for his illustrations. The latter has focused heavily on his embryological plates (Haeckel 1868), which have been a frequent subject of academic debate (see, e.g., Rütimeyer, 1868; His, 1874; Gould 1977; Wells 1999; Richards, 2008; Dombroski, 2003; Watts et al. 2019; Hopwood, 2015). Regrettably, Haeckel’s monumental *Challenger* reports receive little recognition today, widely overshadowed by the aesthetic fame of his *Kunstformen der Natur*. Now, new evidence of a syntype of the deep-sea jellyfish *Atolla wyvillei* Haeckel, 1880 is presented paired with a reexamination and evaluation of Haeckel’s type-description and artistic style of illustrations.

## Materials and methods

### Examined material

Two jellyfish examined in this study were from the collection of Jena (PMJ) and two additional specimens were from Copenhagen (ZMC) (not further listed). All specimens from the PMJ were type-specimens. One is a remaining syntype of *Atolla wyvillei* Haeckel, 1880, PMJ Coel 683 preserved in 70% ethanol. The second was the holotype of *Cyanea annasethe* (Haeckel, 1880) PMJ Coel 860 preserved in 4% formaldehyde seawater solution. Further, two specimens of *A. wyvillei* preserved in 70% ethanol from the ZMC were examined. In 2025, a box containing a microscope slide section of *A. wyvillei* was found in the Ernst-Haeckel-Haus. All 29 slides were investigated to clarify their provenance and Haeckel’s use for the species description.

### Syntype

ANTARCTIC OCEAN • 1 ♀ mature female, 40 mm diameter, 1–4 mm thickness; *Challenger* Station 157, 53°55’S, 108°35’E; depth of 1,950 fathoms; 0.1–0 C°; March 03. 1874; Wyville, T. leg.; dredged; between Kerguelen Island and Melbourne; whole animal; in 70% ethanol; historical ground stopper jar; historical label: “*Atolla Wyvillei* Chall. St. 157. $\:\frac{3}{3}$ 74 1950 F.” with a blue “X” in top right corner [handwritten by Haeckel, E.]; PMJ Coel 683.

### Syntype

ANTARCTIC OCEAN • 29 slides of ♀ mature female, 1874; Wyville, T. leg.; dredged; microscope slide section; embedded in Canada balsam; red staining; historical handwritten annotations on slides; historical cardboard box; labeled: “*Atolla Wyvillei Discomedusae*” [handwritten by Haeckel, E.]; PMJ Coel 1119 (EHH signature: D40100–D40121).

### Holotype

SOUTH AFRICA • 1? undetermined sex, 100 mm umbrella diameter, 40–50 mm umbrella thickness, 90 mm oral arm length; at coasts of Republic of South Africa (Medroide von Südafrika) 1866; Bleek, W. leg.; whole animal; in Formalin (3% formaldehyde and 3% seawater); draped in a ground stopper jar on a nylon thread; PMJ Coel 860.

### Microscope slides restoration

By modern standards, the slides possess an unusual format measuring 47 × 28 mm (± 2 mm), which was one standard from roughly 1868 until at least 1917 (Neuhaus et al. 2017). All sections were embedded in Canada balsam, which displays typical signs of severe aging, including extensive micro-cracking (Schmid et al. 2021). Sadly, these cracks render the specimens almost entirely unusable for high-resolution microscopic examination. The specimens and their coverslips were protected from physical damage during storage by two thick pieces of cardboard affixed with Canada balsam to the top and bottom faces of the glass slides. Most of these cardboard pieces were scattered loosely inside the little box containing the slides. Before cleaning, the cardboard pieces had been sorted to their corresponding slides as far as possible. Therefore, the adhesive residue on the microscope slides and the undersides of the cardboard were used, as matching pairs could be found. Slides and coverslips were cleaned with a bellow, eye cotton (Augenwatte, Kerma Verbandstoff GmbH, Hainichen, Saxony, Germany), distilled water, and a drop of 70% ethanol. Additional smudges were carefully scratched of with a razor blade, broken or loose coverslips were replaced by new ones (Knittel Glass, Braunschweig, Lower Saxony, Germany). Completely cracked objects with loose coverslips were embedded in new natural Canada balsam (Carl Roth GmbH, Karlsruhe, Baden-Württemberg, Germany). For a small subset of slides Xylol (97%) (Carl Roth GmbH) was used to remove the cover slip cleanly and reembed the section. Crumbled pieces of the specimen were reassembled to the extent possible using a spring steel tweezer and a brush with only a few hairs (self-made). All cardboards were glued onto the microscope slide with Tylose MH 300 (Klug-Conservation, Immenstadt, Bavaria, Germany). The cardboard was only cleaned with eye cotton and bellows.

### Photography

Overview pictures were taken with a Canon EOS R5 (Canon, Krefeld, Germany), mounted on a StackShot macro rail (Cognisys, Traverse City, MI, USA) equipped with a Canon MP-E 65 mm f/2.8 1–5× Macro Photo Objective (Canon, Krefeld, Germany). Single focused images were stacked using Zerene Stacker 1.04 (Zerene Systems LLC, Richland WA). For an even illumination, a white plastic dome (Rayher, Laupheim, Germany) was grinded with abrasive paper (Starcke, Melle, Germany) and colored with white (RAL 9010) spray paint (Maston, Veikkola, Finland) and placed above the object. The scene was illuminated by two flashlights ((Phottix Juno Li60 Flash, New York, USA) USA) controlled with two Yongnuo RF603C II wireless triggers (Yongnou, Shenzen, China). The jellyfish were carefully placed in a glass petri dish. To reduce drifting of the specimen while taking images, as well as for the lateral image, a glass device was made to hold the medusa in position. For detailed images, the same setup was used. All pictures were developed using Adobe Lightroom classic (version 11.5), editing and image plates were realized with Adobe Photoshop 2024 and Adobe Illustrator 2024 (Adobe, San Jose, USA).

## Results

### Reexamination of *Atolla wyvillei*

**Fig. 1:**
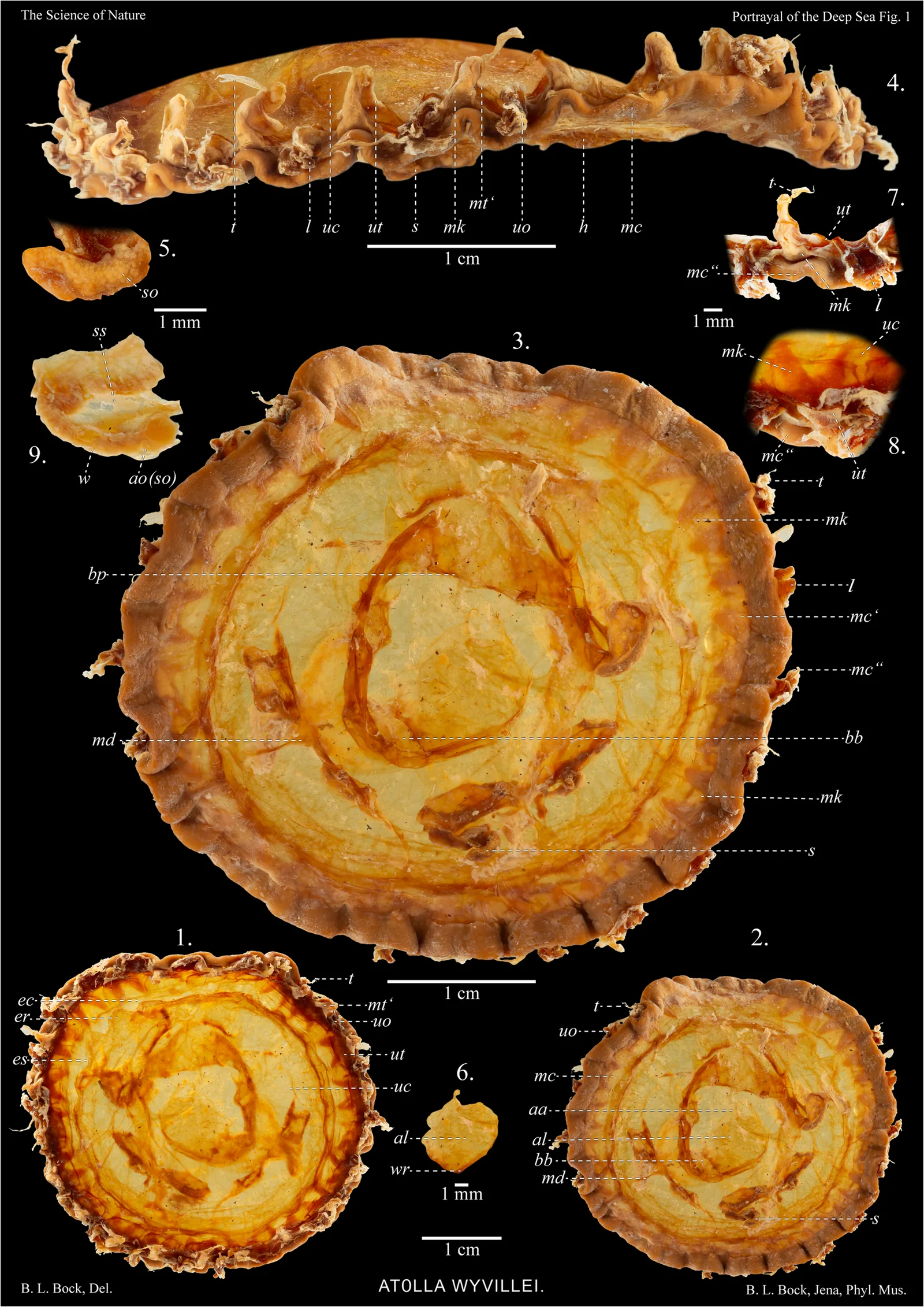
High-resolution images of the syntype of *A*. *wyvillei* (PMJ Coel 683). Plate was arranged to resemble Haeckel’s Deep-Sea-Medusae plate 29. As the specimen is poorly conserved not all characters are clearly visible or could be identified with certainty. To avoid further harm to the valuable object it was decided not to make a section through the tentacle portion. (**1**) Overview from dorsal. (**2)** Ventral overview. (**3**) Magnified ventral view of the whole specimen. (**4**) Lateral view. (**5**) Ovary which is still attached to the specimen, also visible in 2.,3., and 4. as *(s)* (see also Fig. 2b). (**6**) Oral lobe *(al)*, nestled against the ventral part of specimen due to shrinkage. (**7**) Magnified view of tentacle region from lateral. (**8**) Magnified view of tentacle region from dorsal. (**9**) Radial section through ovary. One loosened ovary was cut to perform this image. Lettering follows Haeckel (1881)

To facilitate a rigorous modern comparison, the remaining whole animal has been photographed here for the first time, and a plate has been assembled that models Haeckel’s original Plate 29 of the *Challenger* report as closely as possible (Fig. 1). Additionally, a microscopic slide series from a previously unknown syntype was discovered in the EHH holdings, which was subsequently examined, restored, and photographed.

Three of the five specimens of *A. wyvillei* were captured at Station 157 and possessed an umbrella diameter of 66, 68, and 50 mm at the time of Haeckel’s examination. The remaining two specimens were collected at Station 318 (see Haeckel, 1880 & 1881). All specimens were recorded as mature females (Haeckel, 1880, p. 489), which can at least be confirmed for the remaining type (Fig. 1). Today, the remaining syntype has a diameter of 40 mm, which would represent a shrinkage of 20–41%, compared to Haeckel’s given measurements of Station 157 specimens. It is therefore impossible to determine which one of the three specimens previously examined by Haeckel is the preserved survivor. One can clearly see the artifacts of shrinkage in the tentacles, lobes, umbrella, and gonads. Furthermore, the specimen might have been on the verge of complete dehydration at some point in the last ~ 150 years of storage, which presumably explains why it structurally resembles a potato chip today (Fig. 1).

**Fig. 2:**
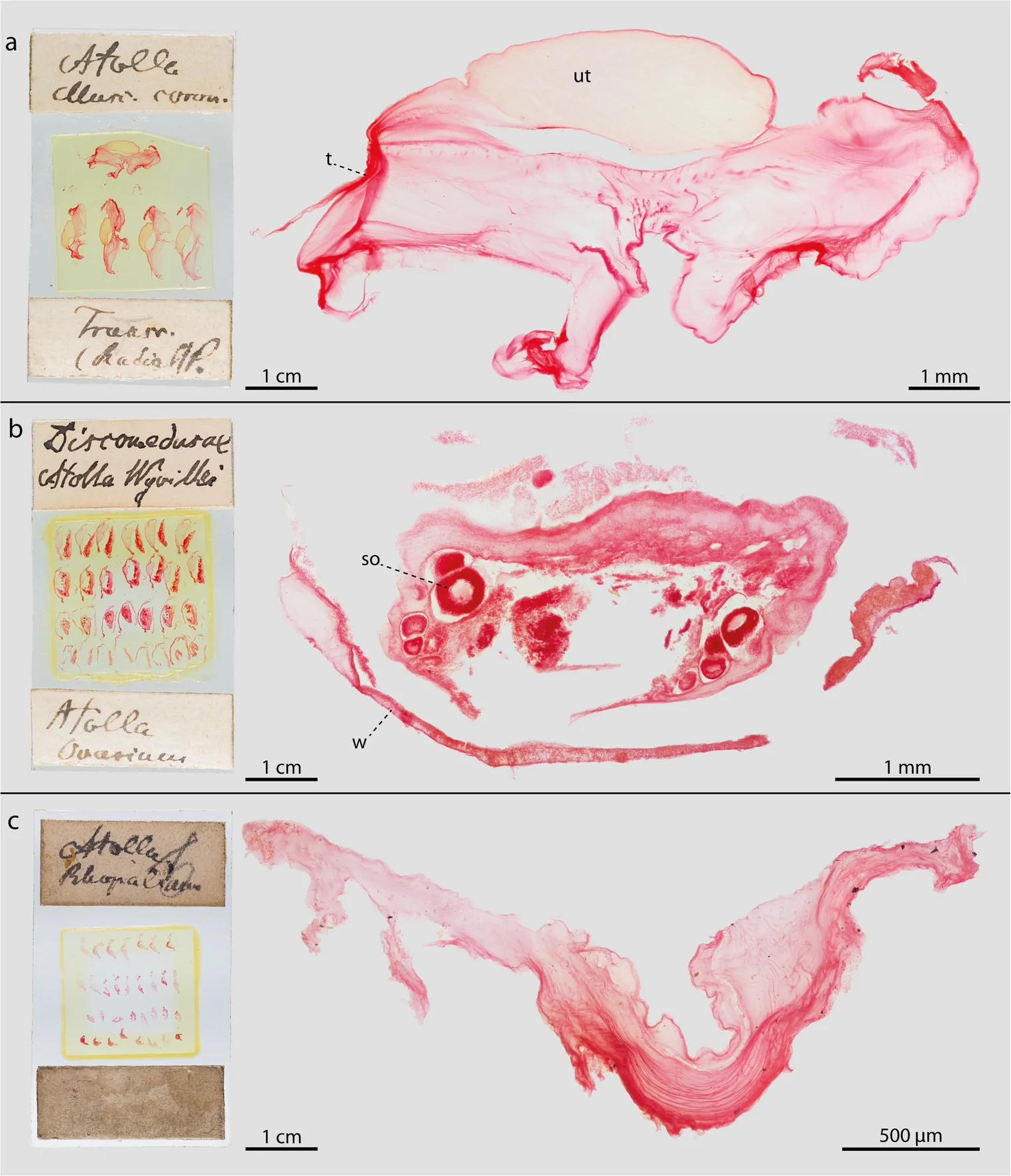
Photographs of three microscope slides from the syntype slide-series of *A. wyvillei* with attached original labels written by Haeckel. (**a**) Transversal orientated radial section through a tentacle pedalia (*ut*) (**b**) radial/tangential section through an ovary with ripped parts of the subumbral wall (*w*) and ova (*so*). (**c**) Radial section through a pedalia of the rhopalia. No rhopalia within the section could be identified

**Fig. 3:**
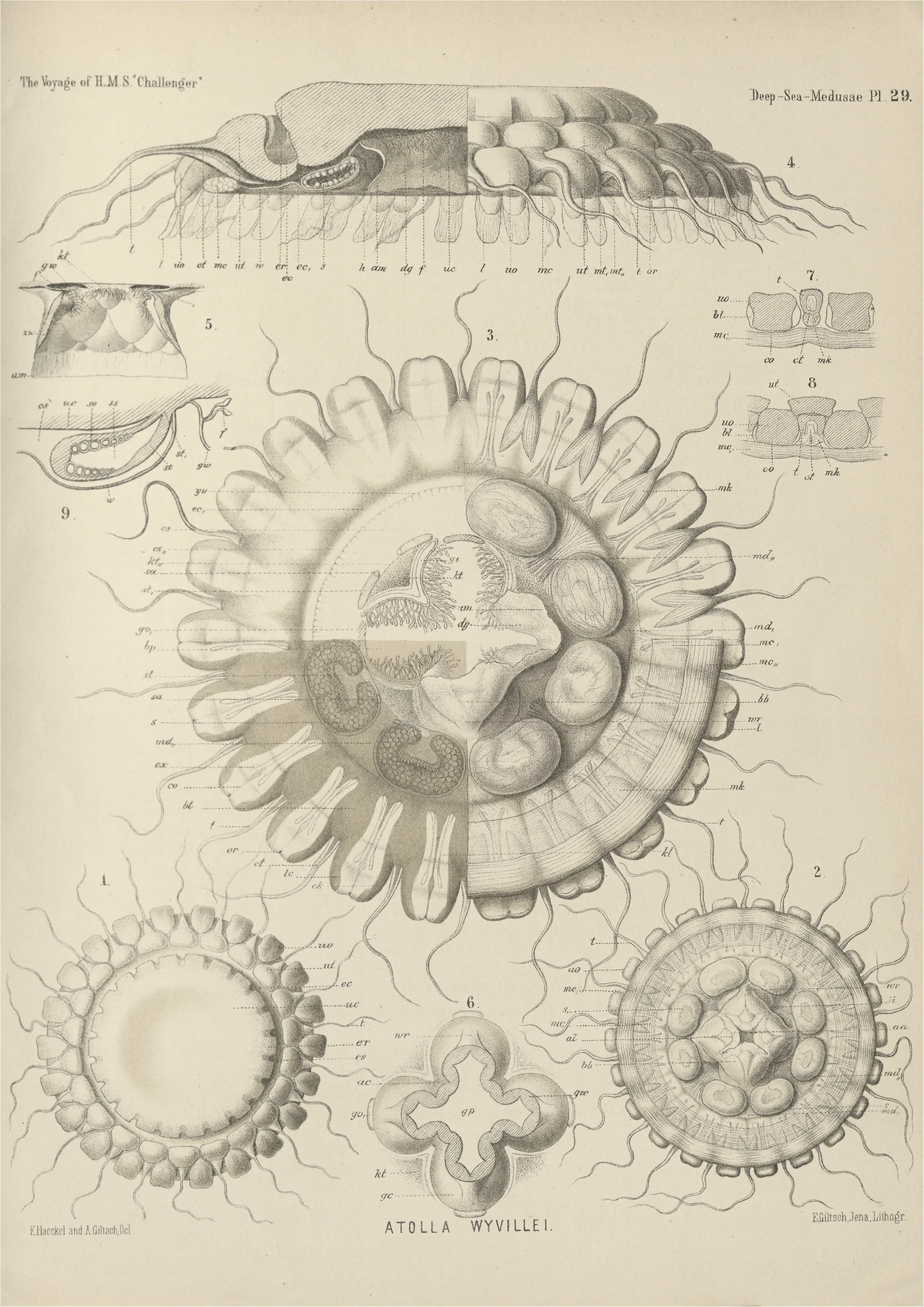
Haeckel and Giltsch’s Challenger Plate 29 of the Report on the Deep-Sea Medusae 1881. (**1**) Overview from dorsal. (**2**) Ventral overview. Both, (**1)** and (**2**) are drawn in natural size which measures 66 mm (measured on an original copy of the report) and could therefore be the whole leftover syntype. (**3**) Subumbral view of the entire Medusa in four quadrants with different layers, twice the natural size. (**4**) Profile view of the entire specimen, twice the natural size. (**5**) Radial section through the oesophagus, twice the natural size (this could not be shown in Fig. 1, nr. 5.). (**6**) Horizontal section through the palatine opening, twice the natural size (only an oral lobe can be shown in Fig. 1, nr. 6.). (**7**) Two tangential sections through a tentacle root and the two adjacent rhopalar pedalia, twice the natural size (no sections were performed on the syntype, only superficial morphology shown in Fig. 1, nr. 7. +8.). (**9**) Radial section through ovary, twice the natural size. (Haeckel 1881)

The historical slide series of the second, previously undocumented syntype was successfully identified. The small storage box was labeled in Haeckel’s handwriting as “Atolla Wyvillei Discomedusae.” and contained 29 histological slides of *A. wyvillei*. With one exception, handwritten notes describing the respective anatomical cuts are present on these cardboard borders (Fig. 2). In general, the preservation state of this slide series is disastrous: most cardboard borders are loosely detached, multiple coverslips are loose, several objects are fragmented, and the slides are heavily smudged and dusty. Only three slides remain in a fair state of preservation, and these happen to be the only recognizably stained histological sections in the entire set (Fig. 2). Whether the superior preservation of the embedding medium on these specific slides stems from the initial chemical fixation and staining process remains speculative, though it matches the visual impression of the series. As noted, Haeckel possessed five ethanol-preserved medusae (representing all specimens known from the genus *Atolla* at the time), meaning these histological sections must have been prepared during his research in Jena. Furthermore, the inscriptions on the box and on two of the slides (Fig. 2b) establish the timeline, as they correspond with Haeckel’s original 1880 classification of *Atolla* within the Discomedusae (Haeckel, 1880, p. 450 ff.), prior to the genus being moved to the order Coronata by Vanhöffen in 1892 (see pp. 20, 24). Additionally, several of Haeckel’s published observations could only have been made using a microscope—specifically via histological sections to view the ovaries, distinct tissue layers (e.g., epidermis, Fig. 2a; mesoglea, Fig. 2a, b; muscle fibers, Fig. 2c), and rhopalia. Haeckel gives credit to Reinhold Teuscher for making the sections, although his remarks regarding the rhopalia are sobering “It was only with considerable trouble that I succeeded in determining their existence […] Their anatomic nature could unfortunately not be found out on account of their small size and the bad preservation of the umbrella margin in all five specimens […]” (Haeckel 1881, p. 116). Haeckel clearly attempted to delineate their anatomical structure, as a physical section of the rhopalar pedalia (*uo*) is present in the collection (Fig. 2c), though he could not resolve it clearly. The sense clubs are indeed tiny and heavily deformed by the historical preservation. Further they lack the ocelli (Jarms et al., 2019), thus it was only possible to confidently identify a single one under the microscope. The number of rhopalar pedalia in the ethanol-preserved type of *Atolla* is 22 (Figs. 1 and 3), which should correspond to the number of sense clubs. No additional sense clubs could be identified within the slide sections, suggesting that the cuts failed to intersect the rhopalia precisely. Some of these historical histological sections are exceptionally thick, easily exceeding 300 μm (e.g., those labeled “Tang. IV”, “flach a”, or “rad. b.”, Table 1). Reviewing his original description for Plate 29, Haeckel mentions a radial Sects. (4, 5, and 9), a horizontal Sect. (6), and two tangential Sects. (7 and 8). These noted orientations perfectly match the handwritten inscriptions on the slide cardboard. Thus, these 29 slides of *A. wyvillei* represent at least one original individual (or parts of it) from the cohort of five specimens Haeckel examined, cementing their status as part of the syntype series. Based on the section series and the preserved whole organism, at least two distinct syntypes of *A. wyvillei* are confirmed to exist today.

**Table 1:** Slides of *Atolla wyvillei* featuring annotations by Haeckel, as recovered from the original repository and categorized according to their respective sections

Microscope slide	Number	annotations by Haeckel
„flach“ (flat)	9	flach I, flach II, flach a, flach b, flach c, flach d, flach e, flach f, flach g
„radial“	4	radial I, radial II, radial III, radial IV
“rad.”	4	rad. a, rad. b, rad. c, rad. d
“Tang.”	4	Tang. I, Tang. II, Tang. III, Tang. IV
“tang”	4	Tang. A, tang b, tang. c, tang. d (bottom), Atolla Wyvillei (top)
-	1	without any labeling
Ovaries	1	Discomedusen Atolla Wyvillei (top), Atolla Ovarien (bottom)
Rhopalia	1	Discomedusen Rhopalien (top)
Tentacle pedalia	1	Atolla alluri.[?] coron. (top), Transv. (Radial)P. (bottom)

Synopsis of *Atola wyvillei* (ordered by year):

Order **Coronata**e Vanhöffen 1892:

Family **Atollidae** Hickson, 1906. [Note 1]

Genus ***Atolla*** Haeckel, 1880. [Note 2]

= ***Collapsis*** Haeckel, 1880 (**junior synonym**).

1. ***A. wyvillei*** Haeckel, 1880. [Type species]. [Note 3]
  = ***C. achillis*** Haeckel, 1880 (**junior synonym**): Fewkes (1886): 8, 934; Vanhöffen (1892): 16.
  = ***A. alexandri*** Maas 1897 (**junior synonym**) is accepted as *A*. *wyvillei* Haeckel, 1880. (see Mayer 1910, pp. 562–567).

1. ***A. vanho***
2. ***A. bairdii*** Fewkes 1886.
3. ***A. verrillii*** Fewkes 1886. [Note 4]
4. ***A. gigantea*** Maas 1897.
5. ***A. chuni*** Vanhöffen 1902.
6. ***A. valdivia***e Vanhöffen 1902. [Note 4]
7. ***A. tenella*** Hartlaub 1909.
8. ***A. vanho effeni*** Russel, 1957.
9. ***A. parva*** Russel, 1958.
10. ***A. russelli*** Repelin, 1962.
11. ***A. clara*** Gershwin & Gordon, 2014.
12. ***A. reynoldsi*** Matumoto, Christianson, Robinson, Haddock & Johnson, 2022.

#### Note 1

Hickson published the family Atollidae in 1906 in *The Cambridge Natural History* (1: 243–424, p. 322). That same year, Henry Bryant Bigelow completed his report on the Medusae and Siphonophorae collected by the US Fisheries steamer *Albatross* in the northwest Pacific, assigning the family name “Atollidae, Bigelow” to the group containing *Collaspis* (Haeckel, 1880) (p. 86). Although authorship is occasionally attributed to Bigelow (e.g., Morandini 2003), his volume was not officially published until 1913.

#### Note 2

The family Atollidae is currently monogeneric. The genus is named after an atoll, Haeckel (1880, p. 488) notes: *Atolla* = Insel mit ringförmigen Korallen-Gürtel (Island with a ring-shaped coral belt). In his *Challenger* report, he wrote: “the corona surrounds the disk as a wall does a fortress, or an atoll, a circular zone of coral reefs, does the island it encloses, from which it is separated by a ring of lagoons” (Haeckel 1881, p. 114).

#### Note 3

Haeckel named the species after Sir Wyville Thomson, who is recorded as the collector of the specimens (Haeckel, 1880, p. 489; Haeckel 1881, p. 113). In the final *Challenger* Reports, the species name is erroneously spelled *Atolla wyvilii*, which represents an incorrect subsequent spelling.

#### Note 4

Both species are doubtful (*nomen dubia*) and could perhaps be *A. wyvillei* (Matsumoto, et al., 2022).

### Diagnosis

The order Coronata is distinguished by the division of the exumbrella into two distinct regions, forming a central circular disc (*uc*) and a marginal zone divided by radiating grooves (*es*) into thickened pedalia (*er*), which terminate at the edge in a circular coronal groove (*ec*) (Matsumoto et al., 2022; see also Haeckel 1881) (Figs. 1 and 2). The family Atollidae is defined by the presence of more than eight rhopalia (which can be difficult to recognize due to their extremely small size) alternating with an equal number of tentacles. Marginal lappets are twice as numerous as the tentacles. Individuals of *A. wyvillei* can reach a bell diameter of up to 150 mm (Kramp 1961), with the margins of the central lens (exumbrella) notched by radial furrows and lacking an annular ridge. The exumbrella surface of the lappets is smooth, and the pedalia are short and broad (Figs. 1 and 3; *uc*,* ec*,* er*,* es*,* uo*; note that *or* is visible only in Fig. 3). The species exhibits a cosmopolitan distribution, though it has not yet been recorded in the Arctic Ocean or the Mediterranean Sea (Haeckel, 1880, 1881; Mayer 1910; Jarms et al., 2019). Relatively little remains known about the deep-sea medusae of the genus *Atolla* (Matsumoto et al., 2022). Haeckel was the first to encounter this enigmatic group on a rigorous scientific level, and to this day, his treatises offer the most comprehensive morphological descriptions available, particularly given that their living behavior was entirely unknown and impossible for Haeckel to observe directly (see Malej et al. 2014). The scarlet-colored, bioluminescent *A. wyvillei* can typically be found at depths ranging from 600 m to more than 3000 m. The species has a hypertrophic tentacle to catch gelatinous prey (e.g. siphonophores), while the other tentacles are used to catch crustaceans and other zooplankton (Hunt and Lindsay 1998). Despite being one of the most prominent medusae of the mesopelagic community worldwide, remarkably little is known about its behavior and life history (Lucas and Reed 2009; Jarms et al., 2019).

## Discussion

### Haeckel and his type specimen

Before discussing his illustration in detail, several historical and nomenclatural contexts require consideration. Haeckel did not designate types in the conventional sense later mandated by the International Commission on Zoological Nomenclature (ICZN) (Aita et al. 2009). Lazarus and Suzuki (2009) argued that because type designation was becoming a standard taxonomic practice during Haeckel’s career, his failure to do so is less excusable (p. 25, presumably referring to the Strickland Code of 1842). However, the ICZN was not founded until 1895, and its first authoritative code was not published until 1905 (ICZN, 1905; Heppell, 1981). Furthermore, the specific physical specimen examined here cannot be considered a holotype, but constitutes a syntype (ICZN Article 73.2), as Haeckel examined five distinct specimens for the species description (see *Monographie der Medusen*, 1880, Vol. 2, pp. 488–489).

Another frequently overlooked aspect addressed in this study is the long-term impact of historical preservation techniques Jones E (2019), which represented a major logistical challenge for the *Challenger* crew. Their standard method was “to plunge the animals direct[ly] from the dredge or trawl into spirit of about 84 to 85 per cent.” (Thomson 1880, p. 24). For larger or highly aqueous specimens, fluid changes were required as the water content of the animal’s body diluted the alcohol. In some instances, absolute alcohol was applied as a final step. It is well established that ethanol preservation causes marine invertebrates, particularly gelatinous jellyfish, to shrink by upwards of 30%, severely distorting the specimen and complicating morphological analysis (Toyokawa 1995; Lüskow et al. 2021). Haeckel himself complained about the state of preservation, noting that “the epithelia being almost entirely wanting” (Haeckel 1881, p. 113, see also p. 115, 116, 123). This epithelial loss routinely occurs due to osmotic changes and shrinkage when transferring a medusa directly from seawater into highly concentrated ethanol (personal observation).

### Comparative visual analysis

**Fig. 4:**
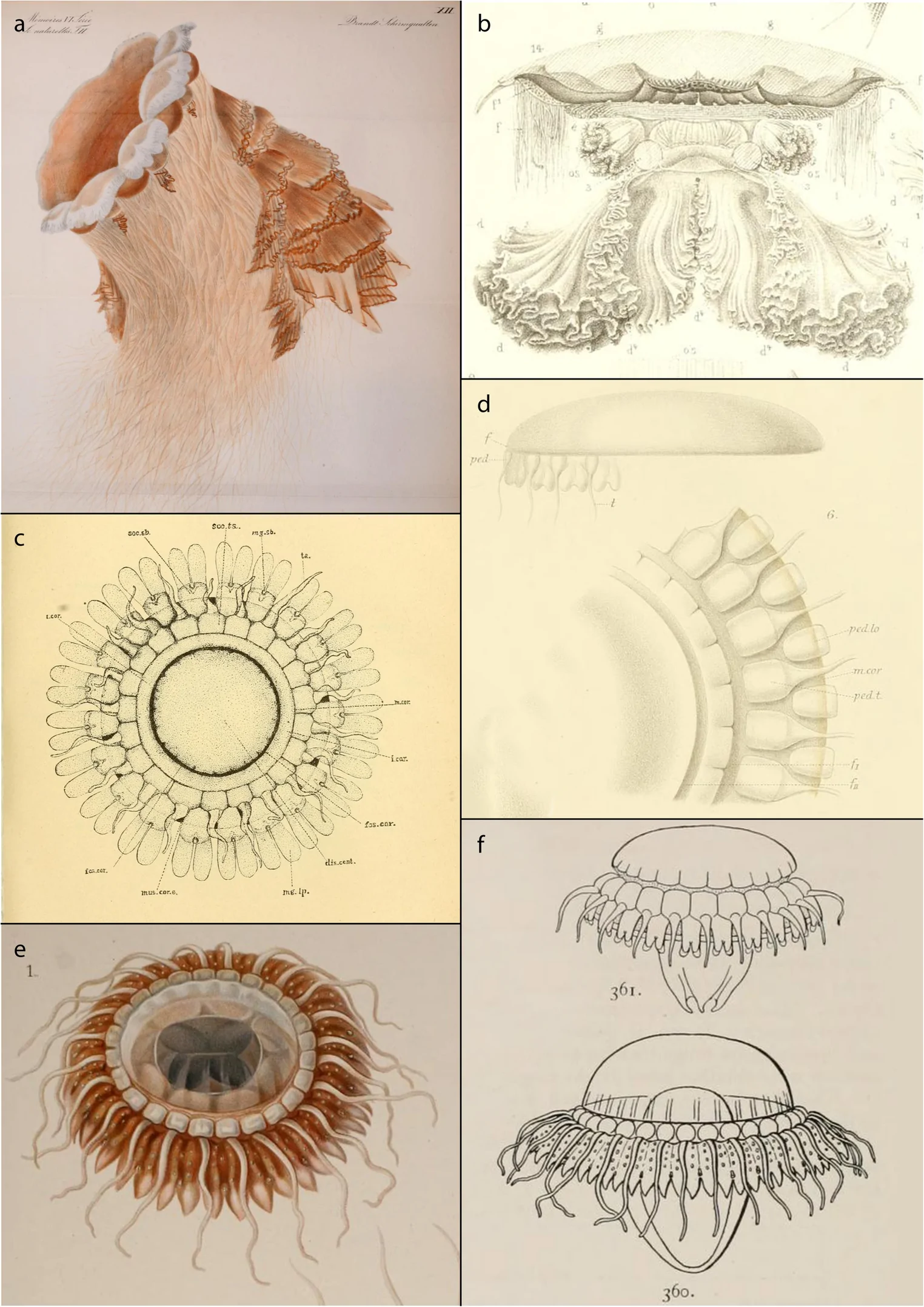
Scientific illustrations of a variety of medusae from six different authors. (**a**) *Cyanea postelsi* (Brandt 1835, pl. XII) drawn by Alexander Postels (1801–1871), habitus. (**b**) *Cyanea* (*arctica*) *capillata* (Linnaeus, 1758) drawn by Antione Sonrel in Agassiz (Agassiz 1860), habitus. (**c**) *A*. *bardii* (Fewkes 1886) by Fewkes, dorsal view. (**d**) *A*. (*alexandri*) *wyvillei* (top), lateral view, and *A*. *gigantea* (bottom), dorsal view by Maas. (**e**) *Atolla chuni* (Vanhöffen 1902, pl. 1.) drawn by Ewald Heinrich Rübsaamen (1857–1919), habitus. (**f**) Habitus line drawings in text of *A. wyvillei* (361. top) and *A*. *chuni* (360. bottom) by Mayer (Mayer 1910)

Comparing the subjects of Haeckel’s illustrations with those of his contemporaries reveals only minor differences. The most significant points of contrast are found in Alfred Goldsborough Mayer’s *Medusae of the World* (Mayer 1910). Obviously, both authors shared a common scope: Haeckel attempted to depict the entire phylogeny of medusae, while Mayer produced a comprehensive treatise on the medusae of the global ocean. In a distinct “Haeckelian” manner, Mayer produced 76 high-quality color plates, which were used primarily to show medusan habitus and coloration, as well as changes in the body during locomotion (e.g., pl. 74). In contrast to Haeckel’s work, however, these plates completely lack lettering (with some exceptions), containing only figure numbers and a scale. Additionally, Mayer utilized photographs to document a variety of color patterns (pl. 70, 71, and 72). The primary divergence from Haeckel is that Mayer relied heavily on line drawings, with more than 400 accompanying his text. Here, a methodological shift toward highlighting the significant traits of a specimen by simplifying and focusing only on key diagnostic features while eliding unnecessary information (Fig. 4f) can be recognized. Such a reductive method was insufficient for Haeckel.

Mayer was not the first to use such drawings for medusae. J. F. Eschscholtz (1793–1831), for instance, presented whole specimens in line-drawn plates (Eschscholtz 1829). Furthermore, Mayer made tracings of drawings by other authors (Nutting 1910), including those of Haeckel (EAH Jena, Mayer AG (1905a), Mayer AG (1905b), Mayer AG (1912)). Nonetheless, no other author utilized them so extensively or was as conscious of presenting information without the distraction of momentarily irrelevant details as Mayer was (at least regarding medusae). The illustrations of other contemporary authors, lithographers, and illustrators do not differ drastically. They can be grouped into categories ranging from the fragile details drawn by Antoine Sonrel (1804–1879) in Louis Agassiz’s contributions to natural history (Agassiz 1860, plate Va) (Fig. 4b) or Haeckel and Giltsch themselves (Fig. 3), to simpler, monochromatic sketches. An example of the latter is the illustration of the second described species of *Atolla* jellyfish, *A. bairdii* Fewkes 1886; by J. W. Fewkes (1850–1930), published six years after Haeckel introduced this enigmatic genus to the scientific world (Fig. 4c; see also Claus 1883). Other authors, such as Otto Maas (1867–1916) and Ernst Vanhöffen (1858–1918), the latter of whom had plates painted by Ewald Heinrich Rübsaamen (1857–1919), continued the established tradition of fine, colored plates in the manner of Haeckel, though their compositions were sometimes less crowded (Fig. 4d, e). While Mayer adopted the traditional approach for his colored plates, he also embraced line drawings adjacent to the corresponding textual information (Fig. 4f). One could argue that certain illustrators were simply more talented than others, but this misses the point that scientific illustrations should be evaluated objectively rather than from a purely artistic standpoint. Drawing is fundamentally an exercise in abstraction, which is why critics sometimes argue that Haeckel should have simply used photography (e.g., Ball 2007). He certainly could have, as could any other scientist of his era, but the process was expensive, logistically difficult (the lighting required to capture a reasonably clear photograph of a gelatinous medusa was a complex challenge), and not yet a widespread practice in the natural sciences (Peres 2008; Belknap 2020).

The plates composed by Haeckel often overflow with detail, delicate structures, and an immense number of letterings, choreographed to almost resemble a Baroque high altar (cf. Maderspacher 2019). This approach represents the natural world in a Renaissance tradition, holding fast to the objective of “scientific realism”, namely the desire to enable the systematic comparison of sets of objects (Moser 2014). Haeckel was driven to capture everything visible and attempting to maintain a selective view. This dual focus is evident in the individual detailed parts positioned alongside overviews of illustrated specimens (Fig. 3 nr. 5., 7., 8., and 9.). This methodology does not render his drawings or descriptions useless by any means (contra Gould 2000; Stephens & Calder, 2006). Scientific illustrations are commonly employed to simplify complexities and act as a universal language among scientists (Briscoe 1996; Cerviño et al., 2015). Haeckel’s *Challenger* plates, however, seem to elude simplification, but to display nature in a manner that borders on the hyper-realistic. Haeckel’s distinctive approach to object representation sets him apart, as his technically flawless, highly naturalistic drawings convey a holistic interpretation of nature, a philosophical worldview he later manifested in his 1899 work, *Die Welträthsel*. Despite Haeckel’s classical approach, the plates transition logically from simple overviews of the specimen (Fig. 3, nr. 1., 2.) to deeper morphological views. For instance, Haeckel occasionally separates a magnified object into four quadrants, each showing a different tissue layer peeled back from superficial to deeper structures (Fig. 3 nr. 3.). All illustrations are dotted or hatched to delineate different tissues, depths, and heights, providing a three-dimensional understanding of the specimen. This spatial clarity is further emphasized by drawings from alternative perspectives (Fig. 3 nr. 4.), that are likewise rendered in cutaway layers. As the reader proceeds to the enlarged, detailed perspectives of individual structures, these features are sliced to reveal their internal anatomy from the viewpoint of the prior overviews (Fig. 3 nr. 5. – 9.). These depictions of medusa plates are structurally similar to those of Sonrel (Fig. 4b), who joined the Swiss naturalist Louis Agassiz (1807–1873) in 1833, though Haeckel’s plates appear more orderly and precise. Paul L. Kramp (1887–1975) once politely observed: “It is not to be denied that Haeckel’s artistic temperament and fertile imagination sometimes led him to construct a detailed description and beautiful drawings from an ugly and mutilated specimen” (Kramp 1955, p. 149). This assessment is validated by the fact that Haeckel did not observe most of his described and illustrated specimens alive, nor do we know the exact state of preservation in which he received them (including *A. wyvillei*). Furthermore, the precise degree of influence that Giltsch exerted on the final plates remains an unquantified point of uncertainty—a limitation that largely applies to the works of other contemporary scientists as well. For these reasons, certain illustrations or components thereof may not entirely correspond to living or fresh individuals (Mayer 1910; Kramp 1955). Undeniably, Haeckel’s “*excellent powers of observation*” (Kramp 1955, p. 149) resulted in reliable, accurate illustrations and descriptions. For example, *A. wyvillei* can be readily identified and distinguished from other species within the genus based on his work (see species diagnosis). Thus, Haeckel’s scientific illustrations in his monographs—exemplified here by *A. wyvillei*—fully conformed to the scientific standards of his era, with seemingly extraordinary aesthetic execution. This unique quality is primarily a product of Haeckel’s and Giltsch’s distinctive style of drawing and their arrangement, that appeals to the eye of the observer. Haeckel was delighted by Carl Heinrich Mertens’s (1796–1830) drawings of Siphonophorae, which he described as “[…] among the best and most life-like representations of the class that have ever been produced” (Haeckel, 1888, preface ii), a sentiment that surely influenced his own illustrations of medusae. This stylistic similarity can be seen, for example, in *Cyanea postelsi* Brandt 1835 (pl. XII), drawn by Alexander Postels (1801–1871) (Fig. 4a), which bears a striking resemblance to Haeckel’s famous *Cyanea (Desmonema) annasethe* (cf. *Kunstformen der Natur*, 1904, Pl. 8).

Logically, some of the characters Haeckel described for a given genus or species have been revised, synonymized, or invalidated as new species and a greater number of individuals have been discovered (see Fewkes 1886, p. 934; Mayer 1910, p. 561), which is standard practice in evolutionary taxonomy. Nevertheless, further systematic investigations into the specimens housed within Haeckel’s underrated monographs are highly necessary, following the examples set in part by Aita et al. (2009) and Jarms et al. (2019). This work will require considerable historical sleuthing, an effort initiated in 2015 with the launch of a centralized database website for historical material from the *Challenger* expedition (Morgenroth et al. 2015). Such historical reexaminations may directly counter long-standing critiques claiming that “types have been lost” (e.g., Straehler-Pohl et al. 2022) or that “Haeckel pictured species he had invented”—the latter echoed by Mayer’s remark: “In fact I have seen Haeckel’s species only in medusæ which I have myself preserved” (Mayer 1910, p. 559).

This study was only able to reevaluate a single species and its type-material described by Haeckel. While this provides concrete physical evidence of his empirical methods, several undeniable constraints remain. Over 150 years of storage have led to varying degrees of physical degradation, such as drying or the displacement of cover slips and color alterations, that restrict optical examination. These observations are further constrained by the fact that only one complete specimen of the former type-series is left, precluding new histological preparations. Another factor is the incomplete archival record, despite the preservation of an immense volume of letters and notes, precise details regarding his exact preparation workflow remain unrecorded. While this study provides a compelling case regarding *A*. *wyvillei*, cautious extrapolation is required when evaluating his broader scientific output. Above all, these efforts can recover invaluable cultural assets and help resolve persistent systematic questions, as historical exchanges of materials have occasionally left important types unmarked and unrecognized in collections (Wheeler and O’Riordan 1969).

## Conclusion and historical legacy

Human perception is inherently subjective and naturally tends to prefer and admire “beauty” as something remarkable. Because no observer can completely escape subjectivity, Haeckel’s illustrations may hold a stronger or lesser aesthetic appeal depending on individual taste. Nonetheless, regarding their primary function as scientific drawings, this subjective aesthetic quality renders them neither superior nor inferior to others. Increasingly, modern discussions surrounding Haeckel’s draftsmanship have been influenced by historical prejudice, largely because his embryological plates sparked immense, and partially justified, negative attention from his lifetime to the present day. Through *Kunstformen der Natur*, Haeckel reached, and continues to do so, not just a small circle of specialized scientists, but a broad global audience. Ironically, many members of the public remain unaware of where these iconic illustrations of marine invertebrates originated from, or that the source material was fundamentally a rigorous scientific work rather than an art book. The present study sets the focus on Haeckel’s contribution to the *Challenger* reports, which enhanced our understanding of the oceans and their organisms. The physical reexamination of *A. wyvillei* type specimen, and the unexpected discovery of the historical microscope slide sections of an additional syntype carry profound scientific and historical significance. The material directly refutes debates that Haeckel relied on fleeting species descriptions or purely “invented” artistic interpretations, instead demonstrating that his illustrations were grounded in histological and morphological examinations. Considering the comprehensive accomplishments of the *Challenger* expedition, which left a permanent mark on modern science, there is a touch of historical humor in John Murray’s original editorial note that he hoped; *“*the complete work may be regarded as an interesting contribution to our knowledge of the ocean, and prove useful to a large number of scientific men, as it is the first attempt to deal systematically with Deep-Sea Deposits, and the Geology of the sea-bed throughout the whole extent of the ocean.” (Murray et al., 1891). The *Challenger* expedition was trailblazing, establishing a kind of structural blueprint for all future oceanographic research cruises. Particularly within the field of biology, the *Challenger* samples represent an indispensable historical collection due to the vast number of new species described therein. It is therefore in the critical interest of the scientific community to identify ‘missing’ type specimens that have not yet been relocated, and to systematically revaluate them alongside existing materials. The discovery underscores the hidden value of smaller institutional archives, whose holdings frequently remain unexamined, but are essential for mapping global biodiversity. One such smaller institution, the Phyletisches Museum in Jena, holds a significant number of specimens across various taxa. Thanks to Haeckel’s prominent historical status, this repository includes vital material from the *Challenger* expedition, including personal gifts from Sir John Murray (1841–1914). To facilitate broader scientific access, an illustrated catalogue of the PMJ’s type collection will be published in the future, enabling researchers to locate and review these specimens while fostering deeper engagement with the public.

## Supplementary Information

Below is the link to the electronic supplementary material.

Supplementary Material 1 (DOCX 23.5 KB)

## Data Availability

No datasets were generated or analysed during the current study.

## Abbreviations used are taken from Haeckel’s *Challenger* report 1881, Pl. 29, and the glossary from p. 142 to 152. Further was taken from Lucas & Reed (2010)

aa: Oral opening
al: Oral lobes
ao: Acidophilic oocytes (mature oocytes)
bb: Buccal pouches (perradial)
bp: Perradial gastral pouches
ec: Coronal furrow of the exumbrella
er: Marginal teeth of the umbrella disk
es: Coronal sinus
l: Marginal lobes
mc: Coronal muscle
mc’: Thinner inner coronal muscle
mc’’: Thicker outer coronal muscle
md: Deltoid muscle
mk: Root muscles of the tentacles
mt’: Abaxial tentacle muscle
s: Ovaries
so: Ova
ss: Genital sinus
t: Tentacles
h: Umbrella-cavity
uc: Central umbrella disk
uo: Pedalia of the rhopalia
ut: Pedalia of the tentacles
w: Subumbrella/ subumbral wall
wr: Mesenteries (perradial)

## Collections acronyms

EHA: Ernst-Haeckel-Archive, Jena, Germany
EHH: Ernst-Haeckel-Haus, Jena, Germany
PMJ: Phyletisches Museum, Jena, Germany
UAJ: University Archives Jena, Jena, Germany
ZMC: Zoological Museum Copenhagen, Denmark
