# Supplementary material for: Portrayal of the deep sea: Haeckel’s Atolla wyvillei, the challenger expedition, and the boundaries of science and illustration
Source: Naturwissenschaften. 2026 Sep 14;113(5):109. doi: 10.1007/s00114-026-02154-8 (PMC13574863; doi:10.1007/s00114-026-02154-8)
Supplement: Supplementary file 1 [file 114_2026_2154_MOESM1_ESM.docx]

**The Science of Nature**

**Portrayal of the Deep Sea: Haeckel’s *Atolla wyvillei*, the *Challenger* expedition, and the boundaries of science and illustration**

Bernhard L. Bock

Friedrich-Schiller-University Jena, Institute for Zoology and Evolutionary Research, Erbertstraße 1, 07743 Jena, Germany, [bernhard-leopold.bock@uni-jena.de](mailto:bernhard-leopold.bock@uni-jena.de)

Taxa named in honor of Sir C. Wyville Thomson. Species described within the same publication by the same author are grouped sequentially; otherwise, entries are arranged alphabetically.

1. ***Abyssascidia wyvillii*** Herdman, 1880. Herdman, W. A. (1880). Preliminary report on the Tunicata of the Challenger expedition. Ascidiidae. Proc. R. Soc. Edinburgh. 10(1), p. 470.
2. ***Abyssothyris wyvillei*** Davidson, 1878. p. 436.
3. ***Dyscolia wyvillei*** Davidson, 1878. p. 436.
4. ***Magellania wyvillei*** Davidson, 1878. p.438. Davidson, T. (1878). Extract from a report to Professor Wyville Thomson, F.R.S., Director of the Civilian Scientiﬁc Staff, on the Brachiopoda dredged by H.M.S. Challenger. Royal Society of London, Proceedings. 27(188).
5. ***Amphicteis wyvillei*** McIntosh, 1885. p. 430, Pl. XXVIIa, fig., 2.
6. ***Branchiomma wyvillei*** McIntosh, 1885. p. 501, Pl. XXXIA, fig., 1–3.
7. ***Ilyphagus wyvillei*** McIntosh, 1885. p. 366, plate XLIV fig. 6, plate XXIIIA figs. 11–14, plate XXXVIA figs. 5–7, plate XXXVIIA fig. 1.
9. ***Laetmonice wyvillei*** McIntosh, 1885. p. 44, PI. VII. fig. 3; PI. IVa. figs. 9-11.
10. ***Pareulepis wyvillei*** McIntosh, 1885. p. 131, Pl. XIX fig. 11, Pl. XX figs. 2–3, Pl. XXIV figs. 2-3, Pl. XXV fig. 11, Pl.XIVA figs. 4-6, Pl. XXXIIA fig. 7. McIntosh, W.C. (1885). Report on the Annelida Polychaeta collected by	H.M.S. Challenger during the years 1873-1876. Report on the Scientific	Results of the Voyage of H.M.S. Challenger during the years 1873–76.	Zoology. 12 (part 34).
11. ***Asteromphalus wyville-thomsonianus*** O'Meara, 1877. O'Meara, E. (1876). On the Diatomaceous gatherings made at Kerguelen's Land by H.N. Moseley M.A., H.M.S. Challenger. Journal of the Linnean Society,Botany, 15(82), p. 57, Pl. 1, fig. 5.
12. ***Asteromphalus wyvillii*** Castracane, 1886. Castracane, F. (1886). Report on the diatomaceae collected by H.M.S.	Challenger during the years 1873-1876. Report on the Scientific Results of	the Voyage of H.M.S. Challenger during the years 1873–76. Botany. 2 (part	4), p. 134, Pl. 5, fig. 6.
13. ***Atolla wyvillei*** Haeckel, 1880. Haeckel, E. (1880). Das System der Acraspeden. 2te Hälfte des Systems der Medusen. Acht Nachträge zur Vervollständigung des Systems. Denkschriften der Medicinisch-Naturwissenschaftlichen Gesellschaft zu Jena. 2. p. 488.
14. ***Benthaster wyvillethomsoni*** Sladen, 1882. Percy, W. (1882). Linnean Society of London. (1865). The Journal of the Linnean Society of London. Zoology (Vol. 16, Issue 91, p. 243). Academic Press [etc.].
15. ***Calyptrophora wyvillii*** Wright, 1885. Wright, E.P. (1885). Alcyonaria. Report on the scientific results of the voyage of H.M.S. Challenger during the years 1873-1876. Narrative. 1(2), p. 690.
16. ***Cnemidaster wyvillii*** Sladen, 1889. Sladen, W.P. (1889). Report on the Asteroidea. Report on the Scientific Results of the Voyage of H.M.S. Challenger during the years 1873–1876. Zoology. 30 (part 51), p. 424.
17. ***Cottunculus thomsonii*** Günther, 1882. Günther, A. (1882). Report on the fishes. In: Tizard, T. H., and J. Murray. Exploration of the Faröe channel, during the summer of 1880, in H. M.'s hired ship "Knight Errant.". Proceedings of the Royal Society of Edinburgh. v. 11, p. 679.

[**Note**]: The fish is said to be named after Sir Wyville Thomson (see Coad & Reist, 2018, p. 442). Yet there is no reference given after whom the species is named (see also Günther, 1887 and Günther, 1878). Sir Wyville should participate the exploration of the Faroe Channel with the “Knight Errant”, but due to is bad health he remained at Stornoway. It seems as there was no other “Thomson” associated, like in the case of the *Challenger* with Wyville Thomson and Frank Thomson, so there is a high chance it is named after Wyville – yet it lacks the “unique identifier” of bearing “Wyville” in its name.

18. ***Culeolus wyville-thomsoni*** Herdmann, 1881. Herdman, W. A. (1881). Preliminary report on the Tunicata of the Challenger expedition. Cynthiidae. Proceeding of the Royal Society of Edinburgh. Vol. 11, p. 84.
19. ***Cypridinodes wyvillethomsoni*** Brady, 1880. p. 160, Pl. XXXIX, fig., 12–17.
20. ***Patagonacythere wyvillethomsoni*** Brady, 1880. p. 82, Pl.	XX, fig. 4, a–f.

Brady, G. S. (1880). Report on the Ostracoda dredged by H.M.S. Challenger during the Years 1873-1876. Report on the Scientific Results of the Voyage of H.M.S. Challenger during the years 1873–76. Zoology. 1 (part 3).

21. ***Cystechinus wyvillii*** A. Agassiz, 1879. Agassiz, A. (1879). Preliminary report on the Echini of the Exploring	Expedition of H.M.S "Challenger". Sir C. Wyville Thomson Chief of Civilian	Staff. Proceedings of the American Academy of Arts and Sciences. new series	6, p. 208.
22. ***Dyscolia wyvillei*** plicata Zezina, 1994. Zezina, O. N. (1994). Recent Brachiopods from Underwater Rises of the Western Part of the Indian Ocean. 129, p. 46.
23. ***Echinophoria wyvillei*** R. B. Watson, 1886. Watson, R. B. (1886). Report on the Scaphopoda and Gasteropoda collected by H.M.S. Challenger during the years 1873-76. Report on the Scientific Results of the Voyage of H.M.S. Challenger during the years 1873–76. Zoology 15 (part 42), p. 408, Pl. XIV, fig., 13.
24. ***Endoxocrinus Wyvillethomsoni*** Thomson, 1872a. Former described by Jeffreys as *Annacrinus wyvillethomsoni* (Jeffreys, 1870) but considered to be a *nomen nudum*; Thomson, 1872a. Thomson, C.W. (1872). Royal Society of Edinburgh. (1834). Proceedings of the Royal Society of Edinburgh (Vol. 7, p. 767). Royal Society of Edinburgh. p. 767.
25. ***Ethmodiscus wyvilleanus*** Castracane, 1886. Castracane, F. (1886). Catalogo delle Diatomee rinvenute da D. Pantanelli nel calcare biancastro friable sovrapposto al bacino di lignite di Spoleto. Processi Verbali Societa Toscana di Scienze naturali di Pisa. Atti. p. 170; pl.	14, fig. 6
26. ***Haliomma wyvillei*** Haeckel, 1878. Haeckel, Ernst. (1878). Das protistenreich. Eine populäre uebersicht über das formengebiet der niedersten lebewesen. Mit einem wissenschaftlichen anhange: System der protisten. E. Günther. p. 44, fig. 31.
27. ***Laetmogone wyvillethomsoni*** Théel, 1879. Théel, H. (1879). Preliminary Report on the Holothuridae of the Exploring Voyage of H.M.S. "Challenger" under Professor Sir C. Wyville Thomson	F.R.S., Part 1, Bihang Till K. Svenska Vet. Akad. Handlingar 5(19). p. 10, Pl. 1, fig. 12–13.
28. ***Metacrinus wyvillii*** P. H. Carpenter, 1884. Carpenter, P. H. (1884). Report on the Crinoidea collected during the voyage of H.M.S. Challenger, during the years 1873-1876. Part I. The Stalked Crinoids. Report on the Scientific Results of the Voyage of H.M.S. Challenger during the years 1873–76. Zoology. 11 (part 32). P. 358, Pl. XLVII, fig. 1–5; Pl. XLVIII.
29. ***Myzostoma wyvillethompsoni*** Graff, 1884. von Graff, Ludwig. 1884. Report on the Myzostomida collected during the voyage of H.M.S. Challenger during the years 1873-1876. Report on the Scientific Results of the Voyage of H.M.S. Challenger During the Years 1873–76, Zoology 10(27), p. 30, Pl. VI., fig., 1–2
30. ***Ophiopyrgus wyvillethomsoni*** Lyman, 1878. Lyman, T., 1878, Ophiuridae and Astrophytidae of the exploring voyage of H. M. S. Challenger, under Prof. Sir Wyville Thomson, F. R. S. Part 1, Bulletin of the Museum of Comparative Zoology at Harvard College 5, No. 7, p.121
31. ***Paratelecrinus wyvilli*** Carpenter, 1882. Carpenter, P. H. (1882). Description of new or little known Comatulae. I. On the species of Atelecrinus and Eudiocrinus. II. The Comatulae of the Hamburg Museum. Journal of the Linnean Society of London (Zoology). 16, p. 492.
32. ***Peniagone wyvillii*** Théel, 1882. Théel, H. (1882). Report on the Holothuroidea dredged by H.M.S.	'Challenger' during the years 1873-76. Part i. Report on the Scientific	Results of the Voyage of H.M.S. Challenger during the years 1873–1876.	Zoology. 4 (part 13), p. 42, Pl. X, fig. 3, 4.
33. ***Platymaia wyvillethomsoni*** Miers in Tizard, Moseley, Buchanan & Murray, 1885. Tizard, T.H., Moseley H.N., Buchanan J.Y. & Murray J. (1885). Narrative of the cruise of H.M.S. Challenger, with a general account of the scientific results of the Expedition. Report on the Scientific Results of the Voyage of H.M.S. Challenger during the years 1873–1876. (Vol. 1, Issue 2, p. 590). Neill.
34. ***Punctulum wyvillethomsoni*** Friele, 1877. Friele H. (1877). Preliminary report on the Mollusca from the Norwegian North Atlantic Expedition in 1876. Nyt Magazin for Naturvidenskaberne, 23, p. 3.
35. ***Thenea wyvillei*** Sollas, 1886. Sollas, W.J. (1886). Preliminary account of the Tetractinellid sponges Dredged by H.M.S.‘Challenger' 1872-76. Part I. The Choristida. Scientific Proceedings of the Royal Dublin Society (new series). 5, p. 184.
36. ***Tricranastrum wyvillei*** Haeckel, 1879. Haeckel, E. (1879). Natürliche Schöpfungsgeschichte: geimeinverständliche wissenschaftliche Vorträge über die Entwickelungslehre im Allgemeinen und diejenige von Darwin, Goethe und Lamarck im Besonderen. p. 705, fig. 5.

Now Haeckel’s described Radiolaria follow, which are all from his *Challenger Report*. Haeckel, E. (1887). Report on the Radiolaria collected by HMS Challenger during the years 1873-76. Report on the Scientific Results of the Voyage of HMS Challenger during the years 1873-76. Page and plate are directly given behind species:

37. ***Amphicraspedum wyvilleanum*** Haeckel, 1887. p. 523, Pl. 45, fig. 12.
38. ***Challengeron wyvillei*** Haeckel, 1887. p. 1660, Pl. 99, fig. 15.
39. ***Castanella wyvillei*** Haeckel, 1887. p. 1683, Pl. 113, fig. 6.
40. ***Dicranastrum wyvillei*** Haeckel, 1887. p. 551, Pl. 47, fig. 3.
41. ***Lichnodictyum wyvillei*** Haeckel, 1887. p. 1231.
42. ***Tuscarora wyvillei*** Haeckel, 1887. p. 17070, Pl. 100, fig. 3, 3a–3c.

***Wyville Thomson Ridge*** 60° 0' 0" N (60°) 7° 15' 0" W (-7.25°), a ridge discovered which was predicted from theoretical considerations. It was named in honor of Sir Wyville, published in 1882.

There are two genera named after Sir Wyville, but both are treated as junior synonyms:

***Wyvillea*** Haswell 1879 accepted as ***Ischyrocerus*** Krøyer, 1838.

***Wyvillethomsonia*** Wright, 1870 accepted as ***Thenea*** Gray, 1867.
